# An ontology for major histocompatibility restriction

**DOI:** 10.1186/s13326-016-0045-5

**Published:** 2016-01-11

**Authors:** Randi Vita, James A. Overton, Emily Seymour, John Sidney, Jim Kaufman, Rebecca L. Tallmadge, Shirley Ellis, John Hammond, Geoff W. Butcher, Alessandro Sette, Bjoern Peters

**Affiliations:** La Jolla Institute for Allergy and Immunology, 9420 Athena Circle La Jolla, San Diego, California 92037 USA; University of Cambridge, Trinity Ln, Cambridge, CB2 1TN UK; Cornell University College of Veterinary Medicine, Ithaca, New York 14853-6401 USA; The Pirbright Institute, Ash Rd, Woking, GU24 0NF UK; The Babraham Institute, Cambridge, CB22 3AT UK

**Keywords:** Major histocompatibility complex, Ontology, MHC, Immune epitope

## Abstract

**Background:**

MHC molecules are a highly diverse family of proteins that play a key role in cellular immune recognition. Over time, different techniques and terminologies have been developed to identify the specific type(s) of MHC molecule involved in a specific immune recognition context. No consistent nomenclature exists across different vertebrate species.

**Purpose:**

To correctly represent MHC related data in The Immune Epitope Database (IEDB), we built upon a previously established MHC ontology and created an ontology to represent MHC molecules as they relate to immunological experiments.

**Description:**

This ontology models MHC protein chains from 16 species, deals with different approaches used to identify MHC, such as direct sequencing verses serotyping, relates engineered MHC molecules to naturally occurring ones, connects genetic loci, alleles, protein chains and multi-chain proteins, and establishes evidence codes for MHC restriction. Where available, this work is based on existing ontologies from the OBO foundry.

**Conclusions:**

Overall, representing MHC molecules provides a challenging and practically important test case for ontology building, and could serve as an example of how to integrate other ontology building efforts into web resources.

**Electronic supplementary material:**

The online version of this article (doi:10.1186/s13326-016-0045-5) contains supplementary material, which is available to authorized users.

## Background

**Fig. 1:**
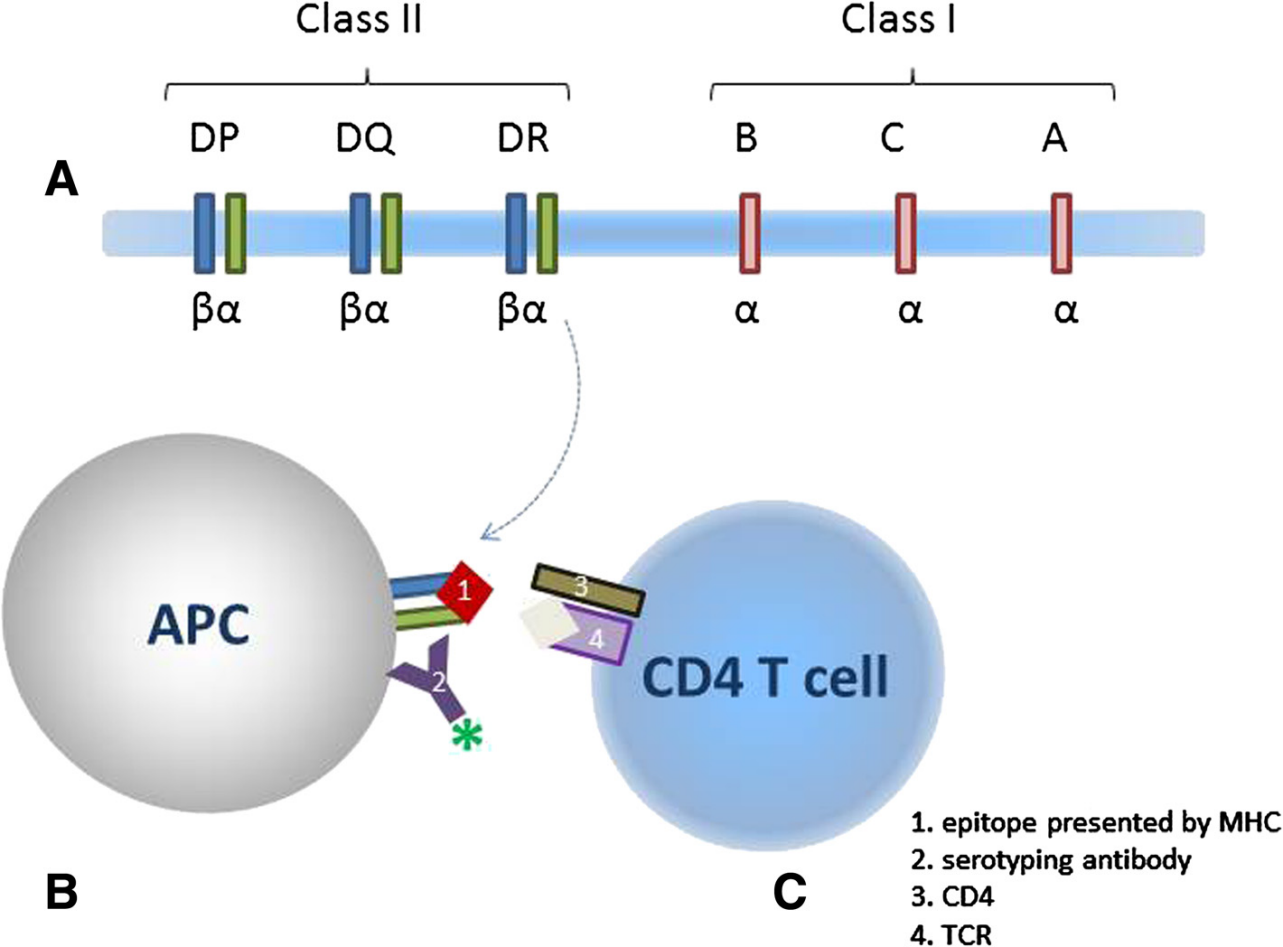
MHC presentation and restriction. **a**. HLA locus of human chromosome 6 encodes specific MHC protein chains. **b**. The MHC on APC presenting epitopes can be bound by antibodies to establish the serotype. **c**. If responding effector cells are known to be CD4 cells, the MHC presenting the epitope can be presumed to be class II restricted

Major histocompatibility complex (MHC) proteins play a central role in the adaptive immune system. First discovered due to their role in transplant rejection, MHC molecules are encoded by a large family of genes with wide variation within each species. MHC molecules typically bind peptide fragments of proteins and display them on the cell surface where they are scanned by T cells of the immune system. If a peptide fragment is displayed by MHC, it can trigger a T cell immune response. Peptides triggering a response are referred to as ‘epitopes’. Thus, binding of epitopes to MHC molecules is an integral step for immune recognition. The specific MHC molecule that presents an epitope to a T cell is knowns as its “MHC restriction”, often called its MHC restriction (or restricting) element. Accurately representing this MHC restriction, which can be determined in different manners, is the goal of the work presented here. Most MHC molecules consist of two protein chains, of which at least one gene is present within the MHC locus. In humans this locus is known as the human leukocyte antigen (HLA) and is depicted in Fig. 1a. There are thousands of different allelic variants of these genes coding for different proteins that result in diverse MHC binding specificities found in the human population. The most precise way of specifying MHC restriction is to identify the exact protein chains that make up the MHC molecule. However, until recently such exact molecular typing was not possible, and patterns of antibody binding were utilized to group MHC molecules together into serotypes that share a common serological (antibody based) recognition pattern, as shown in Fig. 1b. Tying such traditional serotype information together with current sequence based MHC typing techniques is one of the goals of our study. In yet other cases, such as inbred mouse strains, MHC restriction is narrowed down based on the haplotype of the animal, the set of alleles present on a single chromosome and thus expressed consistently together in select subspecies or strains. Another way MHC restriction is sometimes inferred is based on the T cells recognizing the epitope. MHC molecules are divided into three classes: MHC class I, MHC class II, and non-classical MHC. MHC class I molecules present epitopes to CD8+ T cells and are made up of one alpha chain and one β2 microglobulin chain, which is invariant and encoded outside the MHC locus. MHC class II molecules present epitopes to CD4+ T cells and are composed of one alpha and one beta chain, as shown in Fig. 1c. Thus knowing if the responding T cell expresses CD4 verses CD8 can be used to narrow down the possible MHC restriction into classes. At the same time, current research has identified that some T cell populations do not follow this pattern exactly (e.g. some T cells recognizing MHC-II restricted epitopes express CD8). It is therefore important to capture not only the inferred restriction information, but also the evidence upon which it was based.

## Methods

The Immune Epitope Database (www.iedb.org) presents thousands of published experiments describing the recognition of immune epitopes by antibodies, T cells, or MHC molecules [1]. The data contained in the IEDB is primarily derived through manual curation of published literature, but also includes some directly submitted data, primarily from NIAID funded epitope discovery contracts [2]. The goal of the current work was to represent MHC data as they are utilized by immunologists to meet the needs of the IEDB users. We collected user input at workshops, conferences and the IEDB help system regarding how they wanted to retrieve data from the IEDB regarding MHC restriction. These requests were used to identify goals for this ontology project and the final ontology was evaluated if it could answer these requests. As shown in Additional file 1: Table S1, an example of such a request was to be able to query for epitopes restricted by MHC molecules with serotype ‘A2’ and retrieve not only serotyped results but also those where the restriction is finer mapped e.g. to MHC molecule A*02:01 which has serotype A2. We set out to logically represent the relationships between the genes encoding MHC, the haplotypes linking together groups of genes in specific species, and the individual proteins comprising MHC complexes, in order to present immunological data in an exact way and to improve the functionality of our website. Our work builds on MaHCO [3], an ontology for MHC developed for the StemNet project, using the well-established MHC nomenclature resources of the international ImMunoGeneTics information system (IMGT, http://www.imgt.org) for human data and The Immuno Polymorphism Database (IPD, http://www.ebi.ac.uk/ipd) for non-human species. It contains 118 terms for MHC across human, mouse, and dog. We were encouraged by the success of MaHCO in expressing official nomenclature using logical definitions. However, we needed to extend it for the purpose of the IEDB to include data from a growing list of 16 species, as well as data about MHC protein complexes (not just MHC alleles), haplotypes and serotypes. Thus, our current work goes beyond MaHCO, and we have utilized this opportunity to also enhance the integration with other ontological frameworks.

We used the template feature of the open source ROBOT ontology tool [4] to specify the content of our ontology in a number of tables. Most of the tables correspond to a single “branch” of the ontology hierarchy, in which the classes have a consistent logical structure, e.g. gene loci, protein chains, mutant MHC molecules, haplotypes, etc. The OWL representation of our ontology is generated directly from the tables using ROBOT. This method enforces the ontology design patterns we have chosen for each branch, and makes certain editing tasks easier than with tools such as Protégé.

## Results and discussion

Our MHC Restriction Ontology (MRO) is available in a preliminary state at https://github.com/IEDB/MRO. It is based on existing ontology terms, including: ‘material entity’ from the Basic Formal Ontology (BFO) [5], ‘protein complex’ from The Gene Ontology (GO) [6], ‘protein’ from The Protein Ontology (PRO) [7], ‘organism’ from The Ontology for Biomedical Investigations (OBI) [8], ‘genetic locus’ from The Reagent Ontology (REO) [9], ‘has part’, ‘in taxon’, and ‘gene product of’ from The Relation Ontology (RO) [10]. The NCBI Taxonomy was used to refer to each species [11]. Although it is not yet complete, we strive to conform to Open Biological and Biomedical Ontologies (OBO) [12] standards. MRO currently contains 1750 classes and nearly 9000 axioms, including more than 2100 logical axioms. Its DL expressivity is “ALEI”, and the HermiT reasoner [13] completes reasoning in less than 10 seconds on a recent laptop.

Synonyms were also included, as immunologists often utilize synonyms that are either abbreviations or based on previous states of the nomenclature. The current MHC nomenclatures for various species have been revised through several iterations. In order to ensure accuracy and remain up to date with the latest nomenclature, we referred to the well-established MHC nomenclature resources of the IMGT and IPD. For specific species where the literature was most formidable, such as chicken, cattle, and horse, we collaborated with experts in these fields. These experts reviewed the encoded hierarchy by determining whether the inferred parentage hierarchy in their area of expertise reflected their input.

**Fig. 2:**
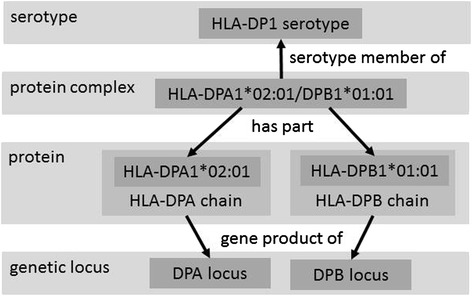
Ontologic relationships between MRO terms

Each MHC molecule for which the IEDB has data is modeled as a protein complex consisting of two chains. Each chain is a gene product of a specific MHC genetic locus. For certain species, sub-loci are also defined, when useful. For example, as shown in Fig. 2 HLA-DPA1*02:01/DPB1*01:01 consists of one HLA-DPA1*02:01 chain, encoded by the DPA sub-locus of DP, and one HLA-DPB1*01:01 chain, encoded by the DPB1 sub-locus of DP. Together these two chains make up one DPA1*02:01/DPB1*01:01 MHC molecule.

**Fig. 3:**
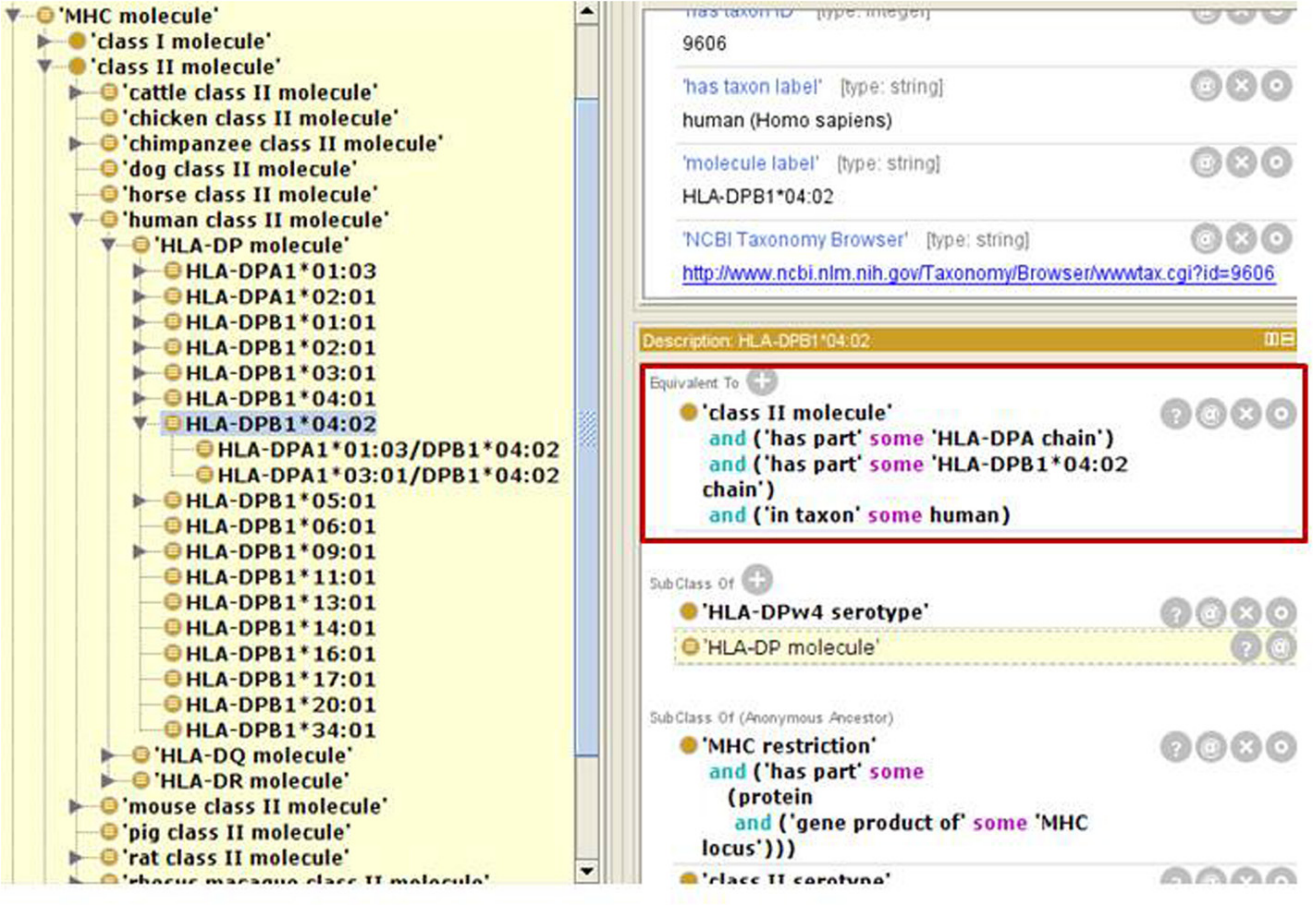
Ontological model showing human MHC class II molecules

When the identity of only a single chain of the complex is known, a “generic” second chain is used to make up the MHC complex. Thus, MHC restriction of HLA-DPB1*04:02 is modeled as one HLA-DPB1*04:02 chain in complex with an HLA-DPA chain that is not further specified, as shown within the context of the hierarchy in Fig. 3.

The data in the ontology drives the Allele Finder on the IEDB website, available at http://goo.gl/r8Tgrz, an interactive application that allows users to browse MHC restriction data in a hierarchical format. We evaluated the ability of MRO to meet the needs of IEDB users, as shown in Additional file 1: Table S1, and found it to meet our initial goals. Currently the use of the ontology is behind the scenes, but we have requested namespace and permanent identifiers from The Open Biomedical Ontologies (OBO). As soon as these identifiers are in place, they will be utilized and displayed on the IEDB website to allow users to link out to the ontology.

In MHC binding and elution assays, the exact MHC molecule studied is typically known; however this is often not the case for T cell assays. When a T cell responds to an epitope, the identity of the MHC molecule presenting the epitope may not be known at all, it may be narrowed down to a subset of all possible molecules or it may be exactly identified. In the context of T cell assays, the MHC restriction can be determined by the genetic background of the host, conditions of the experiment, or the biological process being measured; therefore we represent MHC molecules at a variety of levels and specify the rationale behind the determined restriction using evidence codes.

**Fig. 4:**
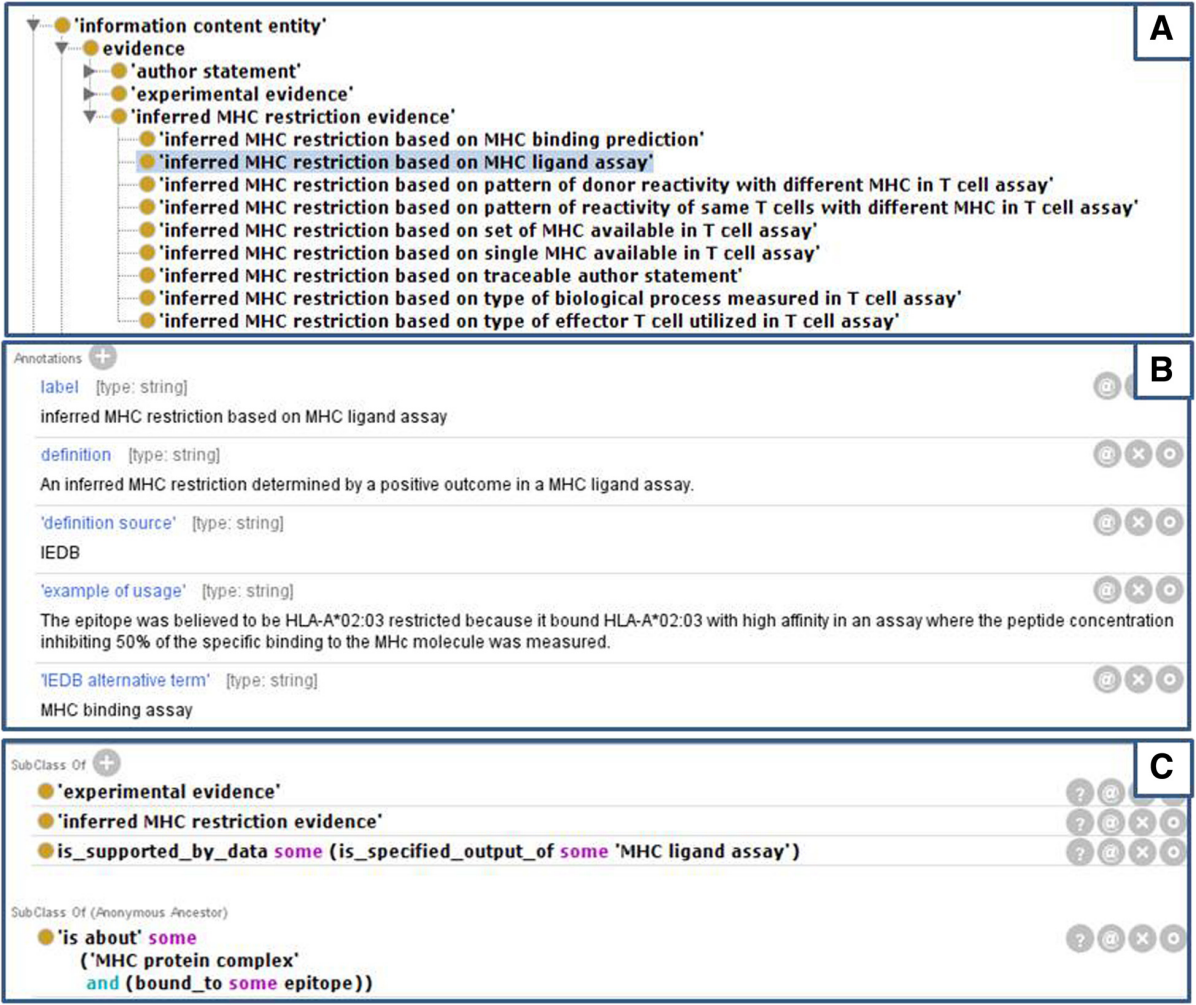
Evidence codes in MRO

As shown in Fig. 4a, IEDB Evidence codes include “author statement” for cases where authors report previously defined restriction and “MHC ligand assay” used for MHC restriction established via an experiment that demonstrated the ability of the epitope to bind strongly to the MHC molecule or to have been eluted from that molecule. Figure 4b shows the metadata associated with this evidence code. “MHC binding prediction” is used when computer algorithms are used to predict the likelihood of an epitope to bind to a specific MHC molecule. In cases where authors analyze the MHC phenotype of a study population and conclude a likely restriction based upon epitope recognition patterns among the subjects, “statistical association” is used as the evidence code. We use a set of evidence codes to communicate restriction shown by the response of T cells to the epitope: MHC complex. These include “Single MHC available” for cases where T cells respond to the epitope when only a single MHC molecule is available and “reactivity of same T cells with different MHC” is used when different APC expressing different MHC are used to narrow the potential restriction. The use of antibodies to block or purify subsets of MHC molecules typically determines restriction to an imprecise level, such as HLA-DR and is conveyed by “set of MHC available.” When the T cells being studied are known to be CD8 or CD4 cells, the restriction can be deduced to be class I or class II, respectively, due to the known binding pattern of the molecules, as depicted in Fig. 1c. This case is communicated by the evidence code of “type of effector T cell.” Lastly, certain T cell responses can indicate the effector cell phenotype of CD8 or CD4, based upon known functions of the subsets and thus, class I or II restriction can be inferred and is noted by the evidence code of “biological process measured.” Figure 4c shows the modeling of these evidence codes in terms of the specific experiments, data transformations performed (using OBI terms), and the type of conclusion drawn. This work is being conducted in parallel with the general alignment of the Evidence Ontology (ECO) [14], which provides succinct codes for such types of evidence, with OBI, which can break down how such a code translates to specific experiments performed.

**Fig. 5:**
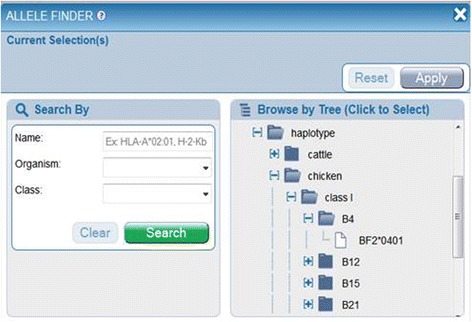
IEDB’s MHC Allele Finder, demonstrating chicken haplotypes

The IEDB MHC Allele Finder application, shown in Fig. 5, now allows users to browse data in different views. MHC molecules are first categorized into ‘class I, class II or non-classical’, and then further subdivided by species. Within each species, MHC molecules are organized by genetic locus. For select species, such as human, there are a large number of MHC molecules known and studied per genetic locus, thus sub-loci are also used in order to present the data in a more user-friendly format. Each MHC molecule is presented under its locus, its haplotype, and/or its serotype, when available, all representing newly added functionalities. The haplotype the host species expresses is represented as immunologists often rely on the known haplotypes of research animals to narrow the potential MHC restriction. For example, when BALB/c (H2d) mice demonstrate a response to an epitope and the responding T cells are CD4+, the restricting MHC can be assumed to be one of the two MHC class II molecules of that haplotype, namely H2 IAd or IEd.

The serotype of an MHC molecule, defined by antibody staining patterns, is relevant in immunology as this was the method of choice to identify MHC molecules until quite recently. In contrast to molecular definitions of MHC molecules based on their specific nucleotide or amino acid sequence, serotyping classifies MHC molecules based entirely on antibody binding patterns to the MHC molecule. These patterns are linked to the panel of antibodies used. Changing the antibody panel changes the serotype of a molecule. This can result in “serotype splits” where MHC molecules that were previously considered identical by one antibody panel, are later found to actually be two different molecules by a different antibody panel. To reflect this extrinsic nature of serotyping, we refer to serotypes as information entities rather than physical entities. Alternatively, the concept of serotype could also be modeled as collections of binding dispositions, but we chose what we thought was the simpler approach. MHC for all 16 species currently having MHC data in the IEDB are modeled to give users the ability to browse the tree in multiple ways and search IEDB data broadly, by entire MHC class, for example, or narrowly by a specific MHC protein chain. As new MHC molecules are encountered, they can be easily incorporated into this ontology.

## Conclusions

In conclusion, we formally represented MHC data building on established ontologies in order to represent MHC restrictions as required by immunologists. Accordingly, we modeled MHC molecules as a protein complex of two chains and established the relationships between the genes encoding these proteins, the haplotypes expressed by specific species, and the MHC classes. Traditional serotype information was also related to specific MHC molecules. Precise MHC restriction was conveyed, as well as inferred MHC restriction and also the experimental evidence upon which the restriction was established. We will continue to formalize this work and will release a completed interoperable ontology later this year. Thus, MHC data in the IEDB is now presented to its users in a hierarchical format which simplifies searching the data and additionally instructs users on the inherent relationships between MHC genes and MHC restriction.

## Additional file

Additional file 1:
**Goals and status of the MRO project.** (XLSX 16 kb)

## Abbreviations

MHC: Major histocompatibility complex
IEDB: The Immune Epitope Database
APC: Antigen presenting cell
HLA: Human leukocyte antigen
IMGT: ImMunoGeneTics
IPD: Immuno Polymorphism Database
MRO MHC: Restriction Ontology
BFO: Basic Formal Ontology
GO: Gene Ontology
PRO: Protein Ontology
OBI: Ontology for Biomedical Investigations
ECO: Evidence Ontology
OBO: The Open Biomedical Ontologies
